# Endotracheal tube defects: Hidden causes of airway obstruction

**DOI:** 10.4103/1658-354X.65123

**Published:** 2010

**Authors:** Khalid Sofi, Kariman El-Gammal

**Affiliations:** *Department of Anesthesia, King Abdul-Aziz Medical City, King Fahad National Guard Hospital, Riyadh, KSA*

**Keywords:** *Endotracheal tube*, *ventilation*, *monitoring*, *intubation*

## Abstract

Manufacturing defects of endotracheal tube (ETT) are still encountered in anesthesia practice. Many such defects go unnoticed during routine inspection prior to their use. Such defects in ETT may lead to partial or complete airway obstruction in an intubated patient. We report a case of partial airway obstruction with a prepacked, single use, uncuffed ETT due to a manufacturing defect in the form of a plastic meniscus at the distal end of the tube. This case report highlights the significance of standard monitoring of ventilation and the role of a vigilant clinician in detecting such defects in avoiding critical events as can arise from the use of such defective ETTs. It also emphasizes the need for double checking ETTs prior to their use.

## INTRODUCTION

Difficulty in ventilating a tracheally intubated patient, as a result of endotracheal tube (ETT) defects, is known in anesthesia practice. Anesthesia gas delivery malfunction, obstruction of breathing circuit and poor pulmonary compliance are common causes which can make ventilation in an intubated patient difficult. Despite the common practice of visual inspection and testing of ETTs for physical defects prior to use, some manufacturing defects still go unnoticed.[1] Obstruction of an ETT is a potentially life-threatening event. Obstruction by mucus, blood or kinking is not uncommon; however, obstruction from a structural defect is also not very rare.[2] When difficulty in ventilation through an ETT is encountered, a quick differential diagnosis is warranted, which includes bronchospasm, pneumothorax, chest wall rigidity and equipment malfunction. We report a case of partial airway obstruction with a 4.5-mm ID uncuffed ETT, due to a meniscus at the distal end of the tube, which was missed on routine inspection prior to use.

## CASE REPORT

A 2-year-old boy weighing 12 kg, ASA 1, with previous urethral injury, was scheduled for an elective urethroplasty. The anesthesia plan included general anesthesia with endotracheal intubation and single shot caudal epidural. The child was premedicated with 5 mg of oral midazolam 1 hour prior to induction. After establishing basic monitoring -Electrocardiography (EKG), pulse oximetry and non-invasive blood pressure, inhalational induction with 5% sevoflurane was initiated followed by establishment of intravenous (IV) access with 22G cannula. Rocuronium 6 mg with fentanyl 20 mcg was given to facilitate endotracheal intubation. Endotracheal intubation with size 4.5 uncuffed PVC ETT (Euromedical, single use) was accomplished without difficulty. Correct placement of ETT was confirmed by end-tidal CO_2_ trace and auscultation. Chest auscultation revealed bilateral but diminished breath sounds and a high resistance was felt at the first manual ventilation. ETT marking at the incisors remained same at 13 cm. The patient was connected to the anesthesia machine, on pressure control mode at an inspiratory pressure of 13 cm H_2_O and a frequency of 20 breaths/minute. The 13 cm H_2_O pressure generated only 30 mL of tidal volume and end-tidal CO_2_ showed an obstructive pattern. There was little chest movement during positive pressure ventilation with no gastric insufflation. Breath sounds were equal but distant. An 8 Fr suction catheter was introduced down the lumen of ETT with resistance. No secretions could be suctioned. Two puffs of aerosolized salbutamol were delivered through ETT for assumed bronchospasm, with no improvement. Oxygenation was maintained at 100% with higher ventilatory pressure. The patient was disconnected from the ventilator and the lungs manually ventilated. However, high resistance to manual ventilation continued. The anesthesia circuit was checked for kinks or obstruction but none was found. The ETT marking at the incisors was same at 13 cm.

**Figure 1:**
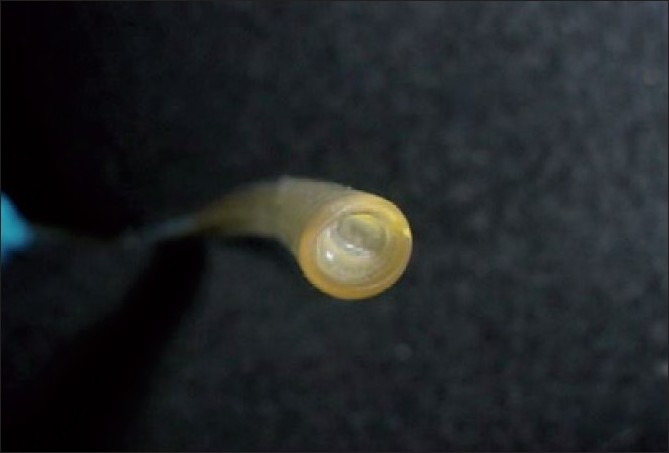
Occlusion of ETT lumen by a plastic meniscus

A possible defect of the ETT was suspected to be the explanation for this clinical scenario. The patient‘s ETT was removed and substituted with another same sized uncuffed ETT with marked improvement in ventilatory parameters. The surgical procedure was accomplished without any further problems. On closer examination of the removed ETT (Euro medical size 4.5 mm ID, PVC uncuffed), it was found that the lumen of the ETT was almost 50% occluded by a plastic meniscus about 7 mm from the distal end [Figure 1]. The defective ETT was sent to the operating room purchase committee for investigation.

## DISCUSSION

After successful endotracheal intubation, ventilation through an ETT may be difficult due to a variety of reasons. The common factors which attribute to difficult ventilation through ETT include anesthesia gas delivery malfunction, obstruction of the breathing circuit (anywhere from common gas outlet to the end of ETT), poor pulmonary compliance (extrinsic or intrinsic), acute severe bronchospasm, tension pneumothorax and endobronchial mass lesion.[3]

A manufacturing defect in an ETT leading to difficulty in ventilation can involve any part of the ETT. Cuff defects leading to herniation of the ETT cuff and intraluminal tracheal obstruction,[4,5] elliptical defects in the wall of the tube causing air leak,[6] kinking of ETT,[7] and intraluminal plastic films and meniscus[8] causing near total airway obstruction have been described. Fatemah *et al*. have described a similar case of ETT obstruction from a manufacturing defect in a 7-mm ID PVC cuffed tube due to a plastic film with a small central perforation covering the distal end of the ETT.[9]

Routine preoperative checking of anesthesia equipment – including the ETT – should be a standard of practice. The tube should be checked for patency of the lumen along with the pilot balloon cuff assembly. In spite of the routine preoperative ETT inspection, some manufacturing defects still go unnoticed and become evident only after initiation of mechanical ventilation through that particular ETT. In our case, although the plastic meniscus at the distal end of the tube was significant, it went unrecognized during preoperative inspection. High resistance on manual ventilation and persistently low tidal volume and high inspiratory pressures during pressure control ventilation lead to the suspicion of ETT defect, as all maneuvers to overcome difficulty in ventilation failed. Besides, the absence of a Murphy's eye in older versions of small-sized ETT, as in our case, made ventilation more difficult. A Murphy eye is an essential part of basic ETT design in current practice.

The aim of highlighting this case report is to ensure double checking of ETTs before their use to avoid unnecessary intraoperative problems that can arise from the use of defective ETTs, compromising patient's safety. Most of the ETT defects described in literature could not be identified during routine preoperative inspection. Thus, the role of a vigilant anesthetist is invaluable for patient safety even in the presence of a defective ETT.

## CONCLUSION

Double checking of anesthesia equipment including the ETT, before its use, along with standard monitoring for ventilation, should be a standard of practice for each patient requiring endotracheal intubation.
